# Quantification of U, Th and specific radionuclides in coal from selected coal fired power plants in South Africa

**DOI:** 10.1371/journal.pone.0229452

**Published:** 2020-05-01

**Authors:** U. A. Q. Ahmed, N. J. Wagner, J. A. Joubert

**Affiliations:** 1 National Nuclear Regulator, Centurion, South Africa; 2 DSI-NRF CIMERA, Department of Geology, University of Johannesburg, Johannesburg, South Africa; Gwangju Institute of Science & Technology, INDIA

## Abstract

Most of South Africa’s energy is derived from the combustion of coal in pulverized coal-fired power plants (CFPP). However, when compared with the rest of the world, limited information regarding the main radioactive elements (U and Th) and specific radionuclides of interest (K^40^, Ra^226^ and Th^232^) from South African CFPP is available in the public domain. This paper aims to quantify the U, Th and specific radionuclides found in the coal used in selected South African CFPP in comparison to world averages found in literature. The U and Th concentrations were obtained by ICP-MS. The main radionuclides, K^40^, Ra^226^ and Th^238^, were quantified using gamma spectrometry. The U concentration and Th concentrations for the coal used in all the power plants was above the world average of 1.9 mg/kg and 3.2 mg/kg respectively. The coals with the highest Th content originated from the Mpumalanga power plant, while the U content in the Freestate power plant samples was the highest of the three. The concentrations of the K^40^ were between 88.43±10.75–110.76±8.92 Bq/kg, which are in-line with world averages of 4–785 Bq/kg. Similarly, the Ra^226^ and Th^232^ values were between 21.69±2.83–52.63±4.04 Bq/kg and 19.91±1.24–22.97±1.75 Bq/kg respectively, which are also in line with the world averages of 1–206 Bq/kg and 1–170 Bq/kg respectively. Radiological hazard indices such as radium equivalent (Ra_eq_); external hazard index (H_ex_) and internal hazard index (H_in_), that were estimated from these average radionuclide concentrations were less than the prescribed values found in literature. This indicated that no significant health risk was posed by the coal being used from these coal fields.

## Introduction

Coal, consisting of a variety of organic and inorganic components, plays an important role in the production of South Africa’s energy, accounting for approximately 77% of electricity production [1]. However, it is the inorganic matter in coal–minerals and trace elements–that have been cited as possible causes of health, environmental, and technological problems associated with the use of coal [2–4]. One reason for health concerns is that toxic trace elements from commercial combustion sources are enriched on the surface of particulate matter (less than 2.5 μm), compared to bulk particulate matter [5]. Some trace elements in coal are naturally radioactive. These radioactive elements include uranium (U), thorium (Th), and their numerous decay products–including radium (Ra) and radon (Rn) [6,7]. Depending on the area from where the coal is mined, it contains varying amounts of radioactive elements [8]. For example, the concentrations of U in coal-hosted ore deposits may vary from tens of ppm to a few thousands of ppm [9]. The global average values for U and Th concentrations in hard coal is 1.9±0.1 mg/kg and 3.2±0.1 mg/kg respectively [10], with Swaine [11] reporting ranges of 0.5–10 ppm for U and Th. According to the International Atomic Energy Agency [12], the average concentration of the main radionuclides in coal are K^40^, 4–785 Bq/kg; Ra^226^, 1–206 Bq/kg and Th^232^, 1–170 Bq/kg.

When coal is burned, coal combustion residuals (CCR) such as fly ash, bottom ash and boiler slag are formed. The concentration of most radioactive elements in solid combustion wastes such as fly ash and bottom ash will be multiple times higher than the concentration in the original coal [13]. For example, in the United States (where the average ash content of coal burned is stated to be approximately 10 weight percent) the concentration of most radioactive elements in solid combustion wastes is approximately 10 times the concentration of the original coal [4]. Therefore, the radionuclides in coal, such as Th^232^, may increase from approximately 10–25 Bq/kg to 200 Bq/kg in the combustion products [14]. The concentration of radionuclides in CCR in South Africa remains unknown, and hence understanding their potential impacts on the surrounding environment is neglected.

Since there is a radiological relationship between the coal fed to CFPP and the resulting CCR [4,13,15,16], there is a need to investigate the coal used in South African CFPP. This analysis begins with respect to the main radioactive elements such as U, Th as well as other specific radionuclides of interest i.e. K^40^, Ra^226^ and Th^232^ for which global averages are readily available. Hence, this paper aims to provide an insight of the coal used in selected South African CFPP in terms of their radioactive contents and related radiological risks in comparison to the averages found globally.

## Materials and methods

### Sample collection and analytical procedure

This study includes coals from three CFPP in different provinces across South Africa; these are in the Mpumalanga, Limpopo and Freestate provinces. The bituminous coal used in these power plants are from a single mine extracting coal from the Witbank, Waterberg and Sasolburg coalfields of South Africa respectively.

Nine samples of the coal used in each power plant were carefully sampled according to ISO 18283:2006 [17] so as to ensure that it was representative of the coal feed. Each of the samples were composite samples from the power utility which were sampled daily and the daily samples composited every 10 days, for a period of 3 months. This ensured homogeneity of the samples. Thus, 27 samples in total were collected and used for radiometric analyses. The samples, each of approximately 2.00 kg, were packed in labelled polyethylene bags that were securely sealed and transported to local ISO accredited laboratories for analysis. Each of the power stations that the samples were obtained from comprises of six production units. A production unit consists of one boiler, a turbine and generator. Each unit has a capacity of 500–665 MW, and thus the installed capacity for these power stations is 3000–3990 MW of power. The ash content of these coals were supplied by the power utility with averages ranging between 25.27% to 40.85% and is typical for CFPP coal qualities in South Africa. The samples are classified as moderate to high ash (Table 1).

**Table 1:** Ash categories of coal from the various coalfields [18].

Location of Coal Fired Power Plant	Ash Yield (%)	Ash Class Category (According to ISO 11760:2005)
**Mpumalanga Province**	25.27% (**≥**20 and <30)	Moderately high ash
**Limpopo Province**	32.84% (**≥**30 and <50)	High ash
**Freestate Province**	40.85% (**≥**30 and <50)	High ash

No specific permissions or permits were required for the collecting of the samples from all three CFPP since authorised personnel from the power stations collected and supplied them.

The received samples were separated into two portions–one intended for ICP analysis and the other for gamma spectrometry. The former portion was milled using a Reutsch mill to obtain a -250 μm sample.

In this study, an Agilent Technologies ICP-MS (7500 Series) was used to determine the U and Th concentrations. XRD-Analytical & Consulting outsourced the ICP analyses to a certified laboratory (UIS Labs) in Johannesburg- South Africa.

Complete digestion of the sample is required when conducting elemental analysis using ICP-MS. This was done by digesting 0.5 g of each powdered coal sample in a PerkinElmer microwave digestion system (Multiwave 3000) using a 5 ml mixture of 65% concentrated HNO_3_ (SpectrosoL grade) and 3 ml of H_2_O_2_ (30%) for 10 minutes. Consequent to ensuring complete digestion of the samples, the solutions were cooled in a water bath, filtered (as per laboratory procedure), and brought up to a volume of 50 ml using ultra-pure water (18.2 MΩ-cm) in a volumetric flask [19]. Thereafter concentrations for U and Th were determined by ICP-MS. The ICP-MS detection limits for U and Th was 0.010 mg/kg. The ICP-MS instrument was calibrated with a series of traceable calibration solutions. The calibration was verified with another set of verification solutions. To verify the accuracy of the analytical technique a SARM (South African Reference Material)—SARM20 was included.

In order to determine the radionuclides K^40^, Ra^226^ and Th^232^ present in the samples, the coal samples were dried for 24 hours in an air-circulation oven at 110°C. Samples were further pulverized to obtain a fine powder and were sieved for homogeneity. Thereafter, 100 g of each sample was placed in plastic containers of 6.5 cm diameter×7.5 cm height, and sealed to make them airtight. The samples were left for a period of 1 month in a designated laboratory cupboard to ascertain the establishment of secular equilibrium between Ra^226^ and Th^228^ with their progeny and to prevent Rn loss. The specific radionuclides of the samples–i.e. K^40^, Ra^226^ and Th232 –were determined using a high-resolution, p-type coaxial HPGe γ-ray spectrometer (Canberra) shielded by cylindrical lead. Similarly described by Khandaker et al. [19], the relative efficiency of the detector was 28.2% and energy resolution of 1.67 keV-FWHM at the 1.33 MeV peak of Co^60^. The gamma spectrometry systems uses the modelling software LabSOCS (Canberra) to determine the efficiency of the geometry used. The detector was coupled to a 16 k MCA to determine the photo-peak area of the γ-ray spectrum and was analysed by Genie 2K software (Canberra) following an ISO accredited procedure. A cylindrical multi-nuclide source was used for detector energy calibration and efficiency determination [20]. The measured detection efficiencies were fitted by using a polynomial fitting function, as described by Khandaker et al. [21] and the fitted efficiencies were used in activity determination of the samples. The minimum detectable activity (MDA) of the γ-ray measurement system at 95% confidence level was calculated according to the procedure by Khandaker et al. [21]. Each sample was counted for 86400 s, and similarly for background counts, in order to obtain the net activity.

### Radiological hazard assessment

The following parameters were evaluated by using the activity concentrations for the radionuclides quantified by gamma spectrometry, that is ^226^Ra, ^232^Th and ^40^K.

#### Radium equivalent activity (Ra_eq_)

In most naturally occurring radioactive material the radionuclides ^226^Ra, ^232^Th and ^40^K are not in secular equilibrium and therefore this one parameter i.e. Ra_eq_ is demarcated in terms of exposure to radiation. The radium equivalent accounts for effective dosage from Rn and its decay products [22]. It is measured in Bq/kg and its definition is primarily based on the assumption that the specific activity of 370 Bq/kg ^226^Ra which is uniformly distributed in any naturally occurring sample may result in an annual effective dosage of 1 mSv at 1 meter above ground level [23]. The Ra_eq_ is quantitatively defined as [24]:

$${Ra}_{eq}=A_{Ra}+{1.43A}_{Th}+{0.077A}_{K}$$ (1)

In the above, A_Ra_, A_Th_, and A_K_ depict the activity concentrations of the respective radionuclides (^226^Ra, ^232^Th and ^40^K). The constants in Eq 1 represents the respective activity conversion rates for ^226^Ra, ^232^Th and ^40^K, which result in the same gamma dose rate at a maximum permissible Ra_eq_ of 370 Bq/kg.

#### External hazard index

The external hazard index (H_ex_) is used for quantifying gamma ray-acquired radiation hazards. The maximum value of 1, corresponding to the radium equivalent’s upper limit at 370 Bq/kg, constitutes the optimum acceptable value for external hazard index [22,25]. Eq 2 is used for computing H_ex_ [24]:

$$H_{ex}=\frac{A_{Ra}}{370}+\frac{A_{Th}}{259}+\frac{A_{K}}{4810}$$ (2)

In the above, A_Ra_, A_Th_, and A_K_ depict the activity concentrations of the respective radionuclides (^226^Ra, ^232^Th and ^40^K). It is assumed that the same rate of gamma dose can be obtained from 4810 Bq/kg ^40^K, 259 Bq/kg of ^232^Th, and 370 Bq/kg of ^226^Ra [26–28].

#### Internal hazard index

Radon and its carcinogenic decay products are hazardous to the respiratory organs [28–30]. The internal exposure to radon and its decay progenies is quantified by the internal hazard index H_in_, which is given by the equation as described by Beretka et al. [24]

$$H_{in}=\frac{A_{Ra}}{185}+\frac{A_{Th}}{259}+\frac{A_{K}}{4810}$$ (3)

In the above, A_Ra_, A_Th_, and A_K_ depict the activity concentrations of the respective radionuclides (^226^Ra, ^232^Th and ^40^K). The values of both H_ex_ and H_in_ must be less than one for radiation hazards to be negligible [14].

## Results and discussion

### U and Th concentrations determined by ICP-MS

Table 2 indicates the average values of Th and U as obtained by ICP-MS for each of the nine samples from the power plants. Both Th and U were present in all the samples. The Th content was higher than that of U, as expected. The highest average Th value (5.66±0.23 mg/kg) was recorded for the Mpumalanga power samples, and the highest average U value (2.91±0.10 mg/kg) was recorded for the Freestate power plant samples. All values recorded are higher than those reported by Ketris and Yudovich [10]. For all the samples, homogeneity was confirmed by comparison of the data from the power utility laboratory daily analyses against that determined on the monthly composite samples, which agreed well. In a parallel study, the monthly composite sample data was compared to the laboratory data for the previous twelve months for each power station, and a good correlation was achieved.

**Table 2:** Th and U concentrations as determined via ICP-MS (mg/kg).

Location of Coal Fired Power Plant	Th	U
**Mpumalanga**	**5.66±0.23**	**2.24±0.09**
**Limpopo**	**5.25±0.31**	**2.62±0.17**
**Freestate**	**4.72±0.11**	**2.91±0.10**
**SARM20 (certified value)**	4.00	18.00
**SARM20 (measured)**	3.95	18.02
**World Averages [10]**	3.20±0.1	1.90±0.1

**Fig 1:**
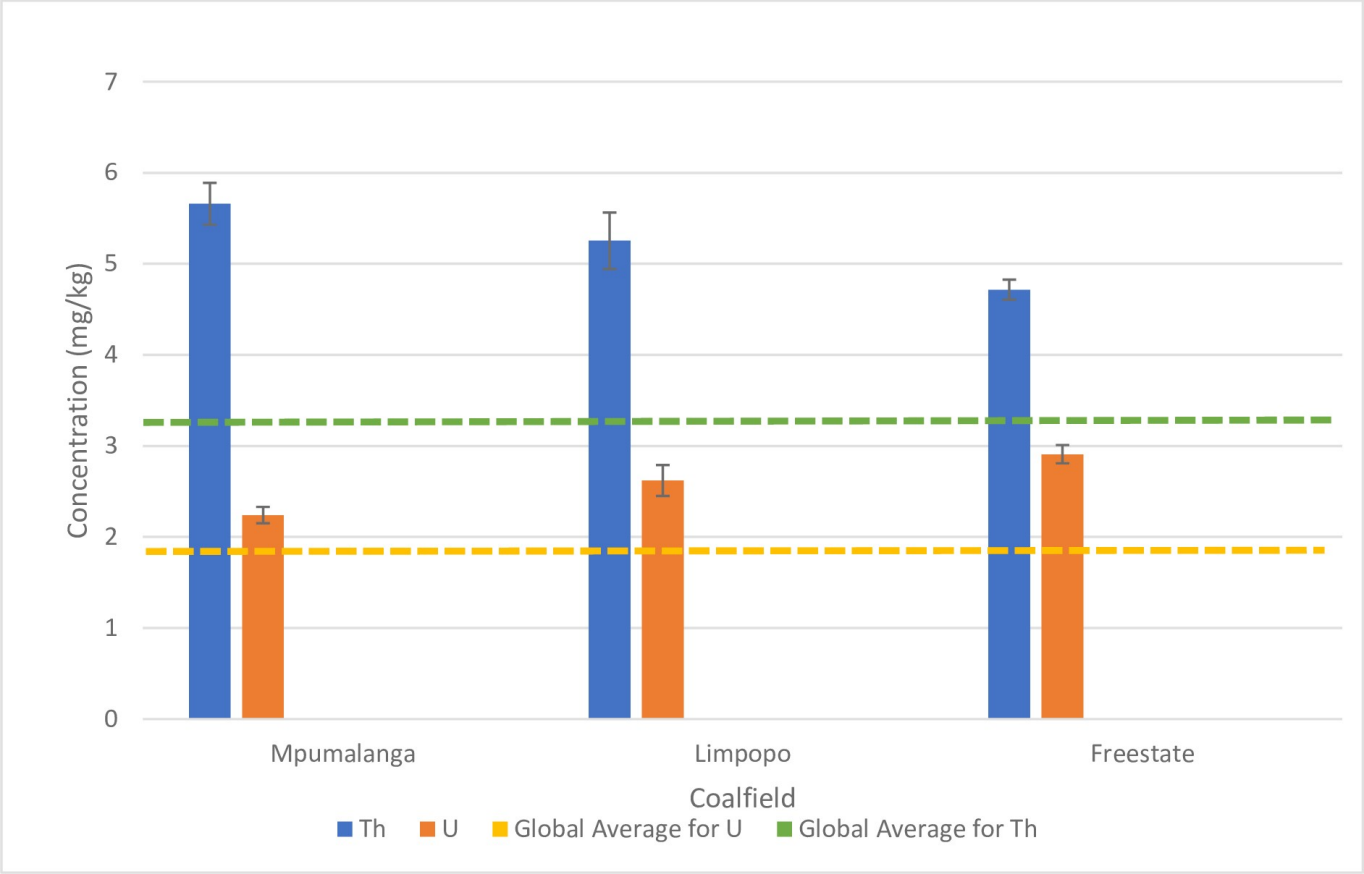
Th and U concentrations reported for the coal used in the three coal fired power plants in relation to the global averages for Th and U i.e. 3.2 ± 0.1 mg/kg and 1.9 ± 0.1 mg/kg respectively- data extracted from Ketris and Yudovich [10].

In Fig 1 it is apparent that the average Th and U concentration in these three South African CFPP are greater than the global average for hard coals [10], but that the values fit in the ranges proposed by Swaine [11] (9.5–10 mg/kg). Wagner and Tlotleng [31] document average values in run-of-mine Waterberg (in the Limpopo province) coals as 3.46 and 5.19 ppm U and Th respectively, which are comparable (bearing in mind the samples used in the current study are beneficiated power station feed coal samples). Bergh et al. [32] documents average U and Th values of 2.6 and 8.9 ppm respectively for some Witbank Number 4 Seam coals (located in the Mpumalanga province), again comparable to the values reported here. Ndhlalose et al. [33] report U values as high as 199 mg/kg with an average of 35,41 mg/kg for coals located in the Springbok Flats Coalfield (located in an isolated basin between the Waterberg and Witbank Coalfields).

### Radionuclides in the coal samples

Table 3 indicates the average values of specific radionuclides (K^40^, Ra^226^ and Th^232^) as obtained by gamma spectrometry for each of the nine samples from the power plants in comparison with coal used by selected countries which were obtained from other studies (indicated in Table 2).

**Table 3:** K^40^, Ra^226^ and Th^232^ radionuclide concentrations for the coal used in three coal fired power plants as determined via Gamma Spectrometry in comparison with world average values and other published works (Bq/kg).

Location of Coal Fired Power Plant	K^40^	Ra^226^	Th^232^
**Mpumalanga (present study)**	**88.43±10.75**	**21.69±2.83**	**22.97±1.75**
**Limpopo (present study)**	**91.29±12.24**	**52.63±4.04**	**21.96±1.54**
**Freestate (present study)**	**110.76±8.92**	**24.39±2.29**	**19.91±1.24**
**World coal averages [12]**	4–785	1–206	1–170
**Hong Kong, China [34]**	24	17	20
**Kolaghat, India [35]**	120.8–151	25.0–49.9	39.3–55.2
**Baoji, China [36]**	99.8	26.3	36.6
**Cayrrhan, Turkey [37]**	123.01	14.55	11.12
**Spain [15]**	104	64	18
**Greece [38]**	108	133	18
**Serbia [39]**	60	16	12
**Kosovo [40]**	36	9	9
**Nigeria-Northeast [28]**	27.38	8.18	6.97

Determination of the specific radionuclides of K^40^ and Ra^226^ indicated that the concentrations in the present study’s samples ranged from 88.43±10.75 to 110.76±8.92 Bq/kg for K^40^ and 21.69±2.83 to 52.63±4.04 Bq/kg for Ra^226^. This is within the world average values for K^40^ (4–785 Bq/kg) and Ra^226^ (1–206 Bq/kg) as reported by the IAEA [12].

Likewise, the average values of Th^232^ is 19.91±1.24 to 22.97±1.75 Bq/kg, and is in line with the world averages reported by the IAEA [12] (1–170 Bq/kg).

The concentrations of K^40^, Ra^226^ and Th^232^ in the coal used in the three South African power plants in the present study tend to compare with coals that have higher radionuclide concentrations of these elements such as those presented in studies of India, China, Turkey and Greece. This may be related to the quality of coal, since the coals fed to these power plants were low quality (high ash) which is (as indicated in Table 2) a typical characteristic of coal used in South African coal fired power plants.

Radiological hazard indices are presented in Table 4 and were determined by using the activity concentrations and Eqs 1–3.

**Table 4:** Radium equivalent (Bq/kg), external and internal hazard index for the coal used in the three coal fired power plants.

Location of Coal Fired Power Plant	Ra_eq_	H_ex_	H_in_
**Mpumalanga**	61.35	0.17	0.22
**Limpopo**	91.06	0.25	0.39
**Freestate**	61.39	0.17	0.23

The calculated value for the Ra_eq_ activity index for the three CFPP were in the range of 61.35 to 91.06 Bq/kg. The H_ex_ values ranged between 0.17 to 0.25. The Ra_eq_ values established were lower than 370 Bq/kg and the H_ex_ was less than 1, both of these values are the prescribed limits set by UNSCEAR [14]. The degree of internal exposure of Rn and its decay products is quantified by the internal hazard index (H_in_) and for this study recorded values of 0.22 to 0.39 which are below the prescribed limits by UNSCEAR [14], which is less than 1. Therefore, the results indicate that there should be no significant health risk due to the coal being used in these CFPP.

## Conclusions

Twenty-seven South African coal samples representative of feed to three different CFPP were quantified in terms of their U, Th and specific radionuclide (K^40^, Ra^226^ and Th^232^) concentrations. Subsequently, the concentrations of U and Th were analysed using ICP-MS and their main radionuclides (K^40^, Ra^226^ and Th^232^) analysed using gamma-ray spectrometry. The samples with the highest Th content in the coal were from the Mpumalanga coal power plant, while the U content in the Freestate coal power plant samples was the highest. The ICP-MS results revealed that all of the samples have a U and Th concentration greater than the global average for hard coals which is 1.9±0.1 mg/kg and 3.2±0.1 mg/kg respectively [10]. The average K^40^ and Ra^226^ obtained from ICP-MS indicated that the concentrations in these samples ranged from 88.43±10.75 to 110.76±8.92 Bq/kg and 21.69±2.83 to 52.63±4.04 Bq/kg respectively, which is within the world average values for K^40^ (4–785 Bq/kg) and Ra^226^ (1–206 Bq/kg) as reported by the IAEA [12]. Likewise, the average values of Th^232^ is 19.91±1.24 to 22.97±1.75 Bq/kg, and is in line with the world averages reported by the IAEA [12] (3.7–97 Bq/kg). The calculated average value for Ra_eq_ were 61.35 to 91.06 Bq/kg. The H_ex_ and H_in_ recorded average values were both below the prescribed value of 1 which is the precautionary limit set by UNSCEAR [41], thus indicating no significant health risk from the coal being used in these three CFPP.

The results obtained in this study lay the groundwork for future research related to the radiological aspects of coal and coal combustion residues associated with CFPP in South Africa.
